# Disulfidptosis-related genes signature predicts prognosis and immune microenvironment in colon cancer

**DOI:** 10.3389/fmolb.2026.1756041

**Published:** 2026-01-28

**Authors:** Wei Jiang, Huaxia Yang, Rui Li, Yanzhi Li, Shaoyou Xia

**Affiliations:** 1 Department of General Surgery, The First Medical Center, Chinese PLA General Hospital, Beijing, China; 2 Department of Biomedical Sciences, Faculty of Health Sciences, University of Macau, Taipa, Macao SAR, China; 3 Department of Digestive Surgery, Xijing Hospital, Air Force Medical University, Xi’an, China

**Keywords:** colon cancer, disulfidptosis, immune cell infiltration, LASSO, WGCNA

## Abstract

**Background:**

Colon cancer (CC), characterized by high incidence and mortality, ranks among the most prevalent digestive malignancies, and reliable molecular tools to predict prognosis and immunotherapy response are needed. Disulfidptosis is a recently identified form of programmed cell death induced by disulfide stress and represents a potential therapeutic target for CC. However, the prognostic and immunological implications of disulfidptosis-related genes (DRGs) in CC remain underexplored.

**Methods:**

RNA sequencing and clinical data from TCGA and GEO databases (GSE39582, GSE17536) were analyzed. A prognostic model including five DRGs (RAB7A, SLC7A11, INF2, FLNA, OXSM) was established using WGCNA, univariate Cox, and LASSO-Cox regression. Patients were stratified into high- and low-risk groups based on risk scores. The model was cross-validated by Kaplan-Meier, time-dependent ROC, nomogra, and multivariable Cox analyses. Immune infiltration (ssGSEA), tumor-mutation burden, miRNA-DRG networks, and drug-sensitivity correlations (CCLE/GDSC/CellMiner) were assessed. Protein expression of RAB7A and OXSM was further examined in tissue microarrays (TMAs) of 97 CCs with matched normal mucosae using immunohistochemistry.

**Results:**

A 5-DRGs prognostic model was established which serves as an independent signal of overall survival and immunotherapy efficacy in CC. Low-risk group patients exhibited an immune-favorable microenvironment and were more abundant with activated CD56 bright NK, γδ-T, and Th17 cells, higher tumor mutational burden (TMB), and elevated CTLA-4 expression, which were likely associated with improved response to immune checkpoint inhibitors (ICIs). As a risk factor, RAB7A relating to poor prognosis was identified in our study, and was a potential target for therapeutic agents such as PLX4720 and PD-0325901, while OXSM was the opposite. Therefore, this model serves as a translatable framework for the precision management of CC, enabling rational risk stratification and facilitating personalized treatment decisions.

**Conclusion:**

The 5-DRGs prognostic model can be used to assess the prognosis of patients with CC, and reflect the characteristics of their immune microenvironment. With RAB7A and OXSM as key determinants, this model provides a clinically applicable framework for risk stratification and personalized treatment guidance.

## Introduction

Colon cancer (CC) is one of the common gastrointestinal tumors and represents highly morbid and lethal. Over 1.9 million new CC cases and 930,000 deaths were reported in 2020 (Morgan et al., 2023). This global burden weighs heavily on China, which faces a substantial and growing number of CC cases. Despite the scale of this public health challenge, limited studies have estimated its risk factors (Zhang et al., 2025). Colonoscopy is the gold standard for CC screening, with high sensitivity and specificity, but it requires experienced physicians and thorough bowel preparation. Therefore, the development of molecular biomarkers needs to be devoted to the detection and treatment of CC. Currently, genomic and transcriptomic studies have provided technical support for investigating biomarkers, including DNA (e.g., mutations, methylation) and RNA (e.g., microRNAs) (Zygulska and Pierzchalski, 2022), and have facilitated the identification of consensus molecular subtypes (Li et al., 2021). These molecular classifications not only offered new approaches for early diagnosis, but also provided ideas for the application of immunotherapy. In recent years, immunotherapy has emerged as a transformative strategy in CC, especially for patients with microsatellite instability-high (MSI-H) or mismatch repair-deficient (dMMR) tumors. Based on the results of the KEYNOTE-177 study, pembrolizumab monotherapy has been widely used as a first-line treatment for patients with unresectable or MSI-H/dMMR colorectal cancer (André et al., 2020). Furthermore, based on data from the CheckMate 8HW study, the dual immunotherapy combination of nivolumab plus ipilimumab can also serve as a treatment option for these patients (Andre et al., 2024). Despite these advances, immune checkpoint inhibitors (ICIs) remain effective only in this molecularly defined minority of cases (Lenz et al., 2022). Therefore, there are necessities to have in-depth researches on the potential regulatory factors and predictive models of CC. The identification of novel molecular markers and immunotherapeutic targets is crucial for expanding the benefits of immunotherapy and improving patient survival rates.

Disulfidptosis offers a new approach to cancer treatment strategies (Liu X. et al., 2023). Koppula et al. have proved that Solute Carrier Family 7 Member 11 (SLC7A11), a solute carrier family member, promotes tumor growth by suppressing ferroptosis and initiating disulfidptosis (Koppula et al., 2021; Zheng et al., 2023). Some bioinformatic analyses have built a bridge between disulfidptosis and cancer, and prognostic models based on disulfidptosis-related markers can predict CC outcomes and reveal potential therapeutic targets (Li Y. et al., 2023). Bioinformatics analyses have further examined disulfidptosis-related markers in relation to the immune microenvironment of CC, leading to the establishment of tumor subtypes (Fan et al., 2020; Xiao et al., 2023). These findings advance CC predictive modeling, yet require experimental validation.

In our study, the co-expression network of disulfidptosis-related genes (DRGs) was reconstructed using weighted gene co-expression network analysis (WGCNA). Then 59 hub genes from the WGCNA model and 29 disulfidptosis-related genes were analyzed using univariate Cox regression to correlate them with survival data. A 5-DRGs prognostic model, including Ras-related protein Rab-7a (RAB7A), SLC7A11, Inverted Formin 2 (INF2), Filamin A (FLNA), and 3-Oxoacyl-ACP Synthase Mitochondrial (OXSM), was established through LASSO-Cox regression analysis, stratifying patients into high- and low-risk groups. We also evaluated the model’s prognostic performance and association with immune cell infiltration, immune checkpoint gene expression, and tumor mutational burden (TMB). Furthermore, immunohistochemical analysis of RAB7A and OXSM was conducted on a 97-case CC tissue microarray (with 83 matched normal mucosae). Long-term follow-up data revealed that elevated RAB7A protein expression correlated with shorter overall survival, while high OXSM expression was associated with more favorable prognosis.

By integrating bioinformatics modeling with tissue microarray validation and overall survival data analysis, we bridge disulfidptosis-related gene signatures with clinical prognosis risk of CC. Our model not only predicts survival but also distinguishes patients with “immune-favorable” microenvironments, suggesting potential utility in identifying candidates for ICIs. We demonstrate that RAB7A and OXSM exhibit contrasting expression patterns and prognostic effects, which are consistently observed at both transcriptional and protein levels, and identifies potential targeted therapeutic approaches, offering translational value for individualized treatment strategies.

## Methods

### Ethics approval

The present study has been approved by the ethics committee of the Shanghai Outdo Biotech Company (Ethics No. SHYJS-CP-230903).

### Data collection

Gene expression and clinical profiles for CC patients were downloaded from The Cancer Genome Atlas (TCGA) database including 487 cases. Normalized array data (GSE39582 and GSE17536, GPL570 Affymetrix Human Genome U133 Plus 2.0 Array, France) and sample annotation files for 556 and 177 cases were obtained from Gene Expression Omnibus (GEO) after removing samples with missing survival information. The baseline information for the enrolled patients is listed in Supplementary Table S1. Besides, The batch effect within two cohorts would be eliminated via the “normalizeBetweenArrays” algorithm of R package “limma” (Ritchie et al., 2015). TCGA-COAD was the training cohort, and GSE39582 was the validation cohort. GSE17536 were added to the analysis when the validation genes were selected.

### Identification of disulfidptosis-related gene set and ssGSEA

DRGs set includes 29 genes obtained from *10.1038/s41598-023-40381-5* (Li J. et al., 2023) (SLC7A11, SLC3A2, NCKAP1, CD2AP, INF2, ACTN4, IQGAP1, DSTN, CAPZB, PDLIM1, ACTB, MYH10, MYL6, MYH9, TLN1, FLNA, FLNB, RAC1, BRK1, CYFIP1, WASF2, ABI2, GYS1, NDUFS1, OXSM, LRPPRC, NDUFA11, NUBPL, RPN1). We estimated enrichment scores of the gene sets for CC patients using the single sample gene set enrichment analysis (ssGSEA) method which was performed by R package “GSVA” (Hänzelmann et al., 2013).

### Constructing gene co-expression network and identification of significant modules

We used WGCNA to reconstruct the co-expression network of DRGs. Firstly, we evaluated the 21776 genes from the TCGA dataset to test their availability and used the R package termed “WGCNA” (Langfelder and Horvath, 2008) to construct a gene co-expression network. Subsequently, we constructed an adjacency matrix to describe the correlation strength between the nodes using the Pearson Correlation. Then, the soft thresholding power (β) value was identified based on the scale-free topology network criterion (signed *R* ^2^) using the WGCNA pickSoftThreshold function and converted the expression matrix into an adjacency matrix. The mean connectivity was calculated across a range of candidate powers (β = 1-30). Based on these diagnostics, β = 5 was chosen as the minimal power at which the signed *R* ^2^ first exceeded 0.85 and reached a plateau while preserving relatively high network connectivity (Figures 2B,C), and was therefore used for subsequent weighted network construction. The module identification was accomplished by dynamic tree cut, whereas the minimum module size was set to 30. Modules with high similarity scores have been combined with a threshold value for each dataset. Finally, Pearson correlation analysis was used to estimate the correlations of the eigengenes in each module with disulfidptosis. The most correlated module was selected as the hub gene module. The next step was to define the constituent genes as DRGs.

### Gene Ontology and KEGG pathway enrichment analysis

Gene Ontology (GO) is a tool for gene annotation that uses a dynamic, controlled vocabulary to classify genes into three categories: biological process, molecular function, and cellular component (Ashburner et al., 2000; The Gene Ontology Consortium, 2019). GO analysis uses different genes to annotate gene functions based on the GO database. The significance levels (P value) and false positive rates (FDR) of each function were calculated using Fisher’s exact test and multiple comparison test to identify the significant functions represented by the differentially expressed genes. The Kyoto Encyclopedia of Genes and Genomes (KEGG) database was used to assign gene sets to specific pathway maps of molecular interactions, reactions, and relation networks (Kanehisa et al., 2017). At present, the KEGG Pathway is categorised into eight sections: overall network, metabolic processes, genetic information transmission, environmental information transmission, intracellular biological processes, biological systems, human diseases, and drug development. The pathway analysis was conducted using Fisher’s exact test and chi-square test for differentially expressed genes to determine the significance of the pathway in which the target gene participated. In this study, we conducted KEGG and GO pathway enrichment analysis using the R package “clusterprofiler” (Wu et al., 2021).

### Survival analysis

59 genes from hub gene model determined by WGCNA analysis and 29 disulfidptosis-related genes were further selected to correlate them with survival data using a univariate Cox regression model. Six candidates (permutation P < 0.1) were inputted into LASSO-Cox regression analysis to construct the prognostic model of these disulfidptosis-related genes with R package glmnet (Simon et al., 2011). Finally, five genes (RAB7A, SLC7A11, INF2, FLNA, OXSM) were included in the prognostic model which was used to calculate each patient’s risk score by a standard formula, which was calculated as the sum of expression values of each disulfidptosis-related genes weighted by their LASSO-Cox coefficients. Using the surv_cutpoint algorithm from “survminer” R package to determine the cut point of risk score, patients from both the training cohort and validation cohort were classified into high- and low-risk groups and the Overall Survival (OS) difference between the two groups was estimated using Kaplan–Meier curves, the log-rank test and Gehan-Breslow-Wilcoxon test depending on situation.

### Nomogram construction

To augment the clinical application value of DRGs risk score signature, we first conducted univariate and multivariate Cox regression analysis integrating risk score and other clinical factors and further established a visualized nomogram model including risk score, TNM stage and Sex index through “rms” (Jr, 2024) and “survival” (Therneau et al., 2024) R package. In addition, we conducted a receiver operating curve (ROC) analysis and used the area under the curve (AUC) values to evaluate the effectiveness of the nomogram in predicting patients’ OS at various time points via R package “timeROC” (Blanche, 2019).

### Gene set enrichment analysis (GSEA)

GSEA was performed based on differentially expressed genes between high and low-risk groups via “clusterProfiler” R package (Wu et al., 2021) to explore the potential molecular mechanisms and to acquire pathways for up and downregulation. A false discovery rate (FDR) of <0.05 was considered statistically significant.

### Immune cells infiltration

R packages “GSVA” (Hänzelmann et al., 2013) was used to perform the ssGSEA algorithm to investigate the immune cell infiltration relationships between the high and low-risk groups. The infiltration of the immune cells in the different groups was visualized using the R package “ggplot2”. The immune cell types analyzed in this study were defined according to a previously published reference, and the corresponding cell-type list is provided in Supplementary Table S2 (Charoentong et al., 2017).

### TMB analysis

TMB creates novel immunogenicity and was previously assumed to predict the effectiveness of ICIs therapy (Hosseini et al., 2020). Mutation profiles of two risk groups were performed using the “MAFTOOLS” R package (Mayakonda et al., 2018) to visualize the frequency and type of mutant genes.

### Prediction of miRNA of DRGs

The Tarbase database (http://www.microrna.gr/tarbase) (Karagkouni et al., 2018) was utilized to predict the associated miRNA of DRGs. The top 10 microT scores of miRNAs involved in each DRGs were extracted and the interaction network was visualized by Cytoscape software (Shannon et al., 2003).

### Drug sensitivity analysis

Drug sensitivity data (IC50 values) integrating three databases CCLE (Barretina et al., 2012), CellMiner (Shankavaram et al., 2009) and GDSC (Yang et al., 2013) were downloaded from the RNAactDrug (Dong et al., 2020) database. Subsequently, the correlation between DRGs and IC50 values of small molecule drugs was calculated and bubble plots were used for databank-by-database presentation.

### Immunohistochemistry (IHC)

The human tissue microarrays (TMAs) were obtained from Shanghai Outdo Biotech Company (HColA180Su23, #XT23-015), including CC samples from 97 patients and adjacent tissues from 83 patients. All 97 tumor samples were taken from primary CC tissues, among which 5 cases were accompanied by metastasis. The histological types included 88 cases of CC, 8 cases of Mucinous adenocarcinoma, and 1 case of Signet-ring cell carcinoma. Following deparaffinization and hydration, antigen retrieval was performed by heating the microarray in sodium citrate solution. The slides were then treated with 3% H_2_O_2_ for 10 min at room temperature to block endogenous peroxidase activity and subsequently incubated in 10% goat serum for 1 h to prevent nonspecific binding. The tissue microarray was incubated overnight at 4 °C with either a primary anti-RAB7A antibody (1:200 dilution, Proteintech, Cat# 55469-1-AP) or a primary anti-OXSM antibody (1:200 dilution, Proteintech, Cat# 16642-1-AP). Immunohistochemical reaction was detected using an Immunohistochemical Detection Kit (Proteintech, PK10006). After dehydration, the microarray was fixed with neutral resin. The staining intensity and area of the microarray were evaluated by three professional pathologists using CaseViewer software.

### Statistical analysis

All statistical analyses were performed using R software 4.3.2 and GraphPad Prism v. 8.01 (GraphPad Software, La Jolla, CA, USA). The Student’s t-test was used to compare values between the test and control groups and P-values <0.05 were considered significant. Based on the determined cut-offs, patients were classified into RAB7A-high/low and OXSM-high/low groups. The association between these categorical variables was analyzed using Fisher’s exact test.

## Results

### Identification of disulfidptosis-related gene modules using WGCNA

**FIGURE 1:**
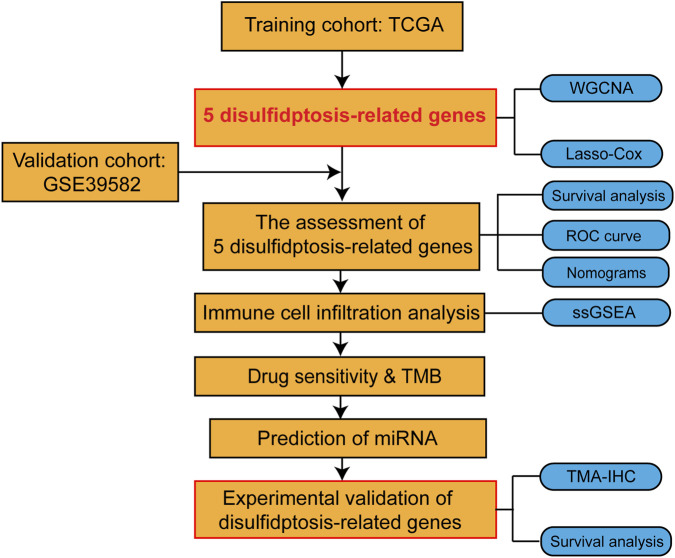
Flow chart of this study design.

**FIGURE 2:**
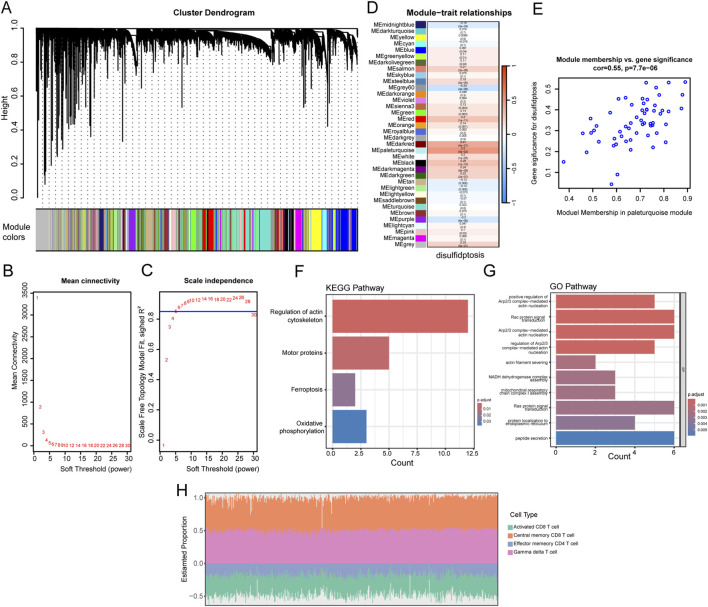
Identification of hub genes model by WGCNA. Dynamic tree cutting algorithms for identification of genes with similar expression patterns in TCGA cohort **(A)**. Analysis of scale independence and mean connectivity to determine the optimal soft-thresholding power β in TCGA cohort **(B,C)**. Heatmap of the correlation between the module eigengenes and disulfidptosis of CRC. We selected the MEpaleturquoise block for subsequent analysis **(D)**. The correlation between gene significance and module membership shown in scatter plot in blue modules **(E)**. KEGG and GO functional enrichment analysis of differential genes in two MEpaleturquoise model **(F,G)**. The proportion of four immune cells built on Mepaleturquoise model genes in TCGA cohort **(H)**.

The flowchart of the whole study is shown in Figure 1. To determine disulfidptosis-related genes, we utilized WGCNA to identify modules correlated with disulfidptosis. The dynamic cut tree was made after merging similar gene modules (Figure 2A). We chose *β* = 5 as an appropriate soft threshold to construct a scale-free network (no scale *R* ^2^ = 0.85) (Figures 2B,C). Among the 35 gene modules, we found that the paleturquoise module containing 58 genes had a close relationship with disulfidptosis fraction features (R = 0.5, P = 5e-32) (Figure 2D). Additionally, the gene in the paleturquoise module was highly correlated with the disulfidptosis status of patients with CC, with a correlation coefficient of 0.55 (P = 7.7e-6) (Figure 2E). Besides, our analysis also correlated these genes from paleturquoise model with metabolic activities and cytoskeleton which were related to disulfide stress–induced cell death (Figures 2F,G). Meanwhile, there were four immune cells enriched in paleturquoise model genes (Activated CD8 T cell, Central memory CD8 T cell, Effector memory CD4 T cell, and Gamma delta T cell) (Figure 2H).

### Construction and evaluation of the five-gene prognostic model

**FIGURE 3:**
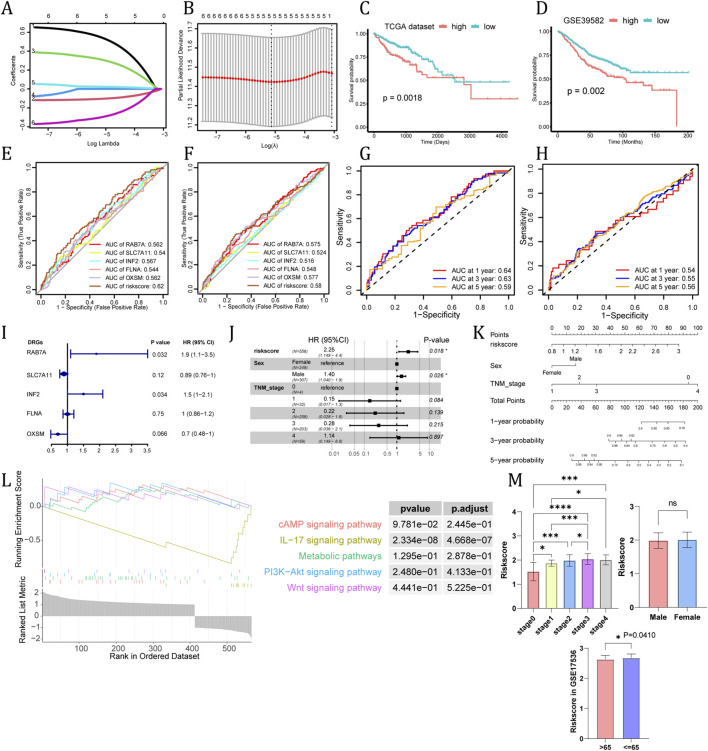
Construct a genetic risk model via DRGs. The trajectory of each independent variable: LASSO-Cox regression analysis of 6 DRGs **(A)**. Cross validation method to select optimal genes **(B)**. Survival curve of high and low risk group in TCGA cohort **(C)** and GSE39582 cohort **(D)**. Clinical correlation analysis and nomogram construction. ROC analysis comparing the performance of DRGs in TCGA cohort and GSE39582 cohort **(E,F)**. ROC analysis comparing the performance of, riskscore for prediction of 1, 3 and 5 years survival probabilities in TCGA **(G)** and GSE39582 cohort **(H)**. Univariate and multivariate Cox regression analysis for DRGs, risk score **(I)** and clinical traits including gender and TNM status **(J)**. Construction of a nomogram based on the risk score and other clinicopathological factors to predict 1-, 3-, and 5-year OS of CRC **(K)**. GSEA analysis for two risk groups **(L)**. Distributions of patients with different TNM stage, sex (in GSE39582) and age (in GSE17536) in high and low risk groups **(M)**.

To assess the prognostic value of hub genes and construct a quantitative measurement for patients’ risk level, univariate Cox regression analysis was performed for TCGA patients and identified six genes closely related to OS, followed by LASSO Cox regression analysis (Figures 3A,B), which finally screened out five eligible genes including RAB7A, SLC7A11, INF2, FLNA, and OXSM. The risk score of each CC sample was calculated via the coefficients of LASSO Cox regression analysis:

$$\begin{array}{ll} risk\;score & =RAB7A\times 0.5424+SLC7A11\times ‐0.1034+\mathrm{INF}2\times 0.3091\\ & \;+FLNA\times 0.0234+OXSM\times ‐0.2975 \end{array}$$

Patients in the training cohort (TCGA cohort) were divided into high-risk and low-risk groups. Kaplan-Meier with log-rank test method was used to observe the difference between the two groups in both training and validation cohorts (TCGA cohort and GSE39582 cohort). The patients in the low-risk group had a longer survival time than those in the high-risk group (P < 0.05, Figures 3C,D). In the TCGA cohort (Figure 3E), the Area Under the Curve (AUC) of the risk score was 0.62, which outperformed or was comparable to the predictive efficacy of most individual genes (e.g., RAB7A AUC = 0.562, SLC7A11 AUC = 0.540, FLNA AUC = 0.544). Similarly, in the GSE39582 validation cohort (Figure 3F), the risk score achieved an AUC of 0.58. Although slightly lower than in the training set, it still demonstrated a comprehensive predictive advantage over single genes (e.g., SLC7A11 AUC = 0.524, INF2 AUC = 0.516), justifying the construction of a multi-gene signature. Furthermore, we assessed the accuracy of the risk model in predicting 1-, 3-, and 5-year survival rates using time-dependent ROC curves. In the TCGA cohort (Figure 3G), the AUC values for predicting 1-, 3-, and 5-year survival were 0.64, 0.63, and 0.59, respectively. In the GSE39582 cohort (Figure 3H), the corresponding AUC values were 0.54, 0.55, and 0.56, respectively.

### Development of the prognostic nomogram and GSEA

Also, we regraded risk score as an independent variable and combined it with other clinical features to perform univariate and multivariate Cox analyses (Figures 3I,J). We further established the prognostic nomogram to quantitatively predict the 1-, 3- and 5-year survival probability of CC patients (Figure 3K). To explore the potential biological processes of the two risk groups, GSEA was performed based on differentially expressed genes. Cyclic adenosine monophosphate (cAMP) signaling pathway, Metabolic pathways, Phosphoinositide 3-kinase−threonine Kinase (PI3K−Akt) signaling pathway, and Wnt signaling pathway were enriched in the high-risk group, while IL−17 signaling pathway was enriched in the low-risk group (Figure 3L). Through comparing risk scores in different stages and genders, we found there was an obvious difference between stage I and stage III but males and females had no significantly different risk scores, which suggests that this model is not gender-specific. Given the absence of age information in the GSE39582 cohort, we found a slightly higher RISK SCORE in GSE17536 for those less than 65 years of age. But it is likely that the difference is limited to a statistical level (Figure 3M).

### Correlation of DRGs expression with tumor immune infiltration

**FIGURE 4:**
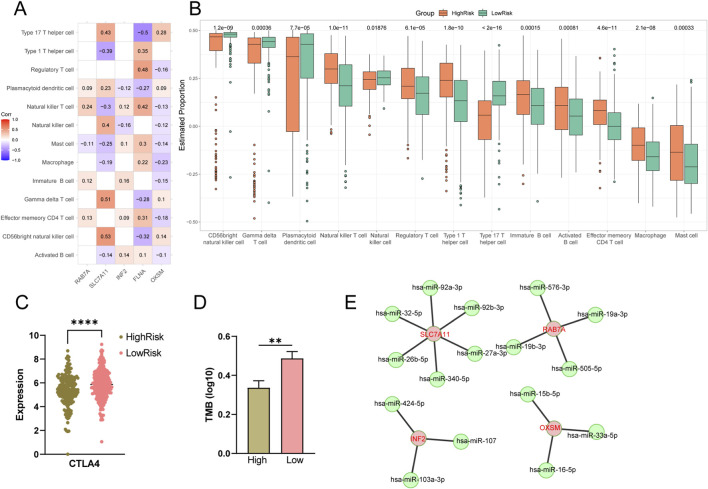
Immune infiltration analysis in high- and low-risk groups. Correlation heat map of 13 types of immune cells and DRGs **(A)**. Bar plot of the proportion of 13 types of immune cells **(B)**. The difference in CTLA4 expression between high- and low-risk patients in the TCGA cohort **(C)**. TMB differences between high- and low-risk patients **(D)**. Interaction networks of DRGs and miRNAs **(E)**.

In this study, we used the ssGSEA method to further analyze the differences between the different risk subgroups of the TCGA cohort in terms of immune infiltrating cells. The significant correlations between the expression of DRGs and the abundance of immune cells were shown by coloured boxes in which the correlation coefficients were also indicated in Figure 4A. We showed the scatterplot of 12 of the most significant pairs of these correlations in Supplementary Figure S1 most of which were associated with SLC7A11 and FLNA. As shown in Figure 4B, CD56 bright natural killer cells, Gamma delta T cells (γδ T), Natural killer cells, Plasmacytoid dendritic cells, Type 17 T helper cells were more abundant in the low-risk group, while Activated B cells, Effector memory CD4 T cells, Immature B cells, Macrophage, Mast cells, Natural killer T cells, Regulatory T cell and Type 1 T helper cells were more abundant in the high-risk group. Our results also suggested that low risk patients exhibited upregulations of CTLA4 in the TCGA cohort (Figure 4C), as well as higher TMB values (Figure 4D). The predicted miRNA interaction networks of RAB7A, SLC7A11, INF2, and OXSM are shown in Figure 4E. These miRNAs have been reported to be involved in immune mechanisms (Supplementary Table S3).

### Genetic and transcriptional characteristics of DRGs combined with TMA analysis

**FIGURE 5:**
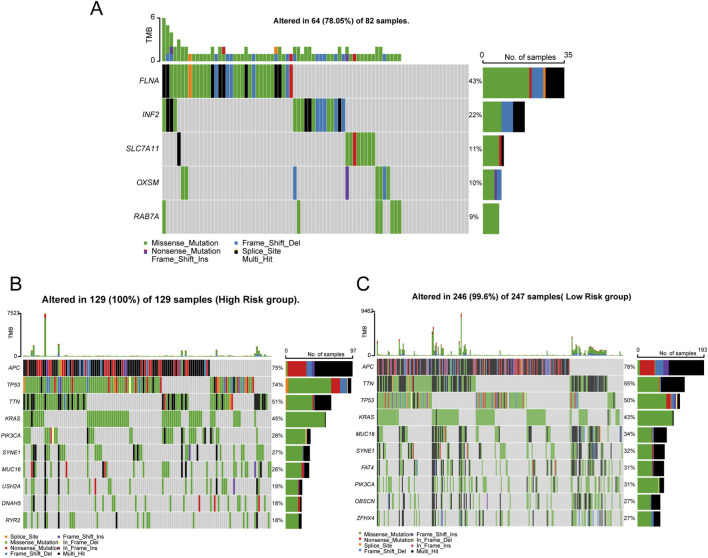
Genetic and transcriptional characteristics of DRGs. Waterfall plots depicting 14 DRGs mutation landscape **(A)**. The 10 high-ranking genes with the highest mutation frequency in the high risk **(B)** and low risk groups **(C)**.

**FIGURE 6 F6:**
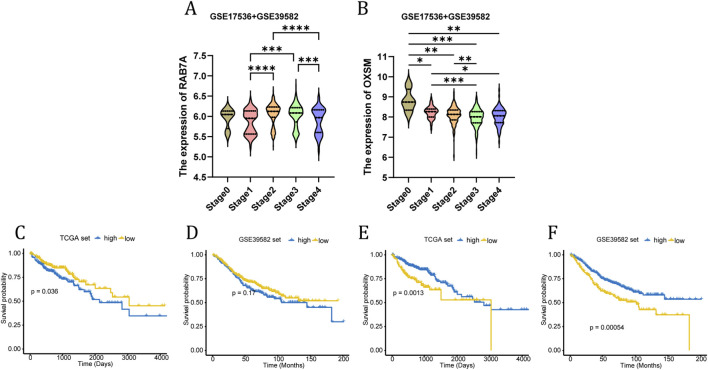
Selection of genes to be validated. The expression of RAB7A **(A)** and OXSM **(B)** differences for patients with different tumor stages. Kaplan–Meier plots showing the differences in OS between patients with high-expressed RAB7A and low-expressed RAB7A in TCGA cohort **(C)** and GSE39582 cohort **(D)**. Kaplan–Meier plots showing the differences in OS between patients with high-expressed OXSM and low-expressed OXSM in TCGA cohort **(E)** and GSE39582 cohort **(F)**.

**FIGURE 7:**
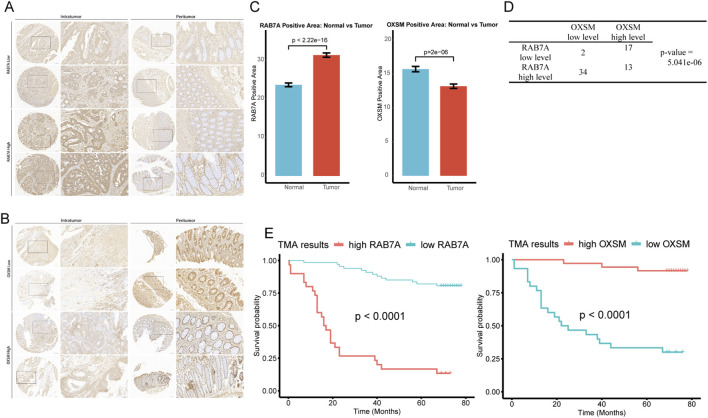
Validation of RAB7A and OXSM through TMA analysis. Representative IHC images of RAB7A **(A)** and OXSM **(B)** in human CRC tissue microarray sample. Percentage of positive area bar plot **(C)**. Fisher’s exact test shows the correlation of RAB7A and OXSM expression in human CRC TMA specimens **(D)**. Kaplan–Meier survival curves of overall survival duration based on RAB7A and OXSM expression in the TMA containing CRC cases **(E)**.

The mutation of DRGs is shown in Figure 5A. The genetic analysis revealed that 64 of the 82 samples (about 78.05%) carried mutations in the regulator of disulfidptosis. Besides, between the high- and low-risk groups, seven of the top ten genes in terms of mutation rate were identical, while the differences were USH2A, DNAH5, and RYR2 in the high-risk group and FAT4, OBSCN, and ZFHX4 in the low-risk group (Figures 5B,C). In addition, we also found that there are few reports on the mechanism studies of RAB7A and OXSM in the CC. The expression of RAB7A was gradually increased from TNM stage I to IV (Figure 6A), but a opposite trend is reflected in the expression of OXSM (Figure 6B). Based on the expression of RAB7A and OXSM, CC patients were categorized into high and low expression groups. The ability of RAB7A expression to discriminate survival outcomes was only demonstrated in the TCGA cohort (Figure 6C) not in the GSE39582 dataset (Figure 6D). For OXSM, the overall survival of high-expression patients was better than that of low-expression patients in both the training cohort and validation cohort (log-rank P = 0.0013 and P = 0.00054, Figures 6E,F). We calculated the positive area of RAB7A and OXSM in CC patients using IHC on TMA. The baseline characteristics of the patients are summarized in Table 1. TMA analysis images showed RAB7A and OXSM expression patterns (Figures 7A,B). Quantitative analysis of IHC staining reveals significantly increased positive area of RAB7A in tumor tissues compared to normal adjacent tissues. OXSM was on the contrary. (RAB7A: P < 2.22e-16; OXSM: P = 2e-06, Figure 7C). A significant positive correlation was observed between RAB7A and OXSM expression levels in tumor samples (P = 5.041e-06, Fisher’s exact test, Figure 7D). Kaplan-Meier survival analysis demonstrates that high expression of RAB7A significantly associated with poor overall survival in CC patients but high expression of OXSM was a protective factor of better overall survival in CC patients (Figure 7E). Therefore, these results demonstrate at both the genetic and protein levels that RAB7A and OXSM have opposing roles in CC.

**TABLE 1:** Basic information characteristics of patients in the colon cancer tissue microarray cohort.

Variables	Case no. (n = 97)	Survival months (mean ± SD)	*p*-value
Age (years old)			0.502
<69	57	52.09 ± 26.61	
≥69	40	57.36 ± 25.00	
Gender			0.881
Female	47	54.06 ± 27.03	
Male	50	54.39 ± 25.19	
Location			0.156
Ascending colon	44	56.95 ± 25.36	
Transverse colon	5	60.20 ± 27.11	
Descending colon	9	60.33 ± 25.26	
Sigmoid colon	37	49.38 ± 26.66	
Unknown	2	—	
Tumor diameter (cm)			0.405
<4.0	13	61.00 ± 23.76	
≥4.0	79	54.01 ± 25.97	
Unknown	5	—	
Pathological classification			<0.001
Adenocarcinoma, not otherwise specified	88	54.10 ± 25.74	
Signet-ring cell carcinoma	1	19.00 ± 0.00	
Mucinous adenocarcinoma	8	60.00 ± 28.20	
Pathological grading			0.037
I	6	73.00 ± 3.16	
II	67	56.06 ± 24.16	
III	23	44.71 ± 31.04	
IV	1	17.00 ± 0.00	
T stage			0.004
T1	1	76.00 ± 0.00	
T2	10	62.50 ± 24.12	
T3	53	59.98 ± 23.83	
T4	33	42.00 ± 26.25	
N stage			<0.001
N0	63	61.95 ± 21.62	
N1	25	42.24 ± 28.14	
N2	9	30.88 ± 24.93	
M stage			<0.001
M0	92	56.08 ± 25.39	
M1	5	20.60 ± 3.91	
Cancer stage (AJCC)			<0.001
1	10	70.00 ± 10.92	
2	51	62.04 ± 21.75	
3	31	41.30 ± 28.16	
4	5	20.60 ± 3.91	

### Drug sensitivity analysis

**FIGURE 8:**
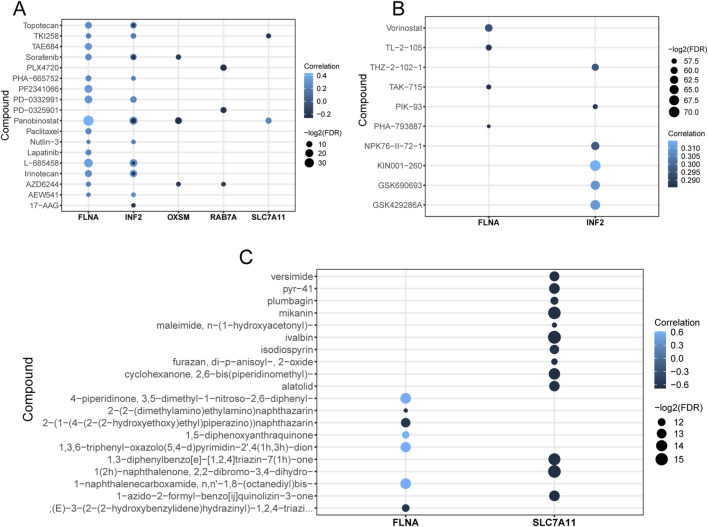
Drug sensitivity analysis. Drugs significantly associated with DRGs from CCLE **(A)**, GDSC **(B)** and CellMiner **(C)** databases.

Given the crucial role of chemotherapies in CC patients’ management strategy, we searched the suitable anti-tumor drugs for five DRGs respectively from the Cancer Cell Line Encyclopedia (CCLE), the Genomics of Drug Sensitivity in Cancer (GDSC) and CellMiner databases. Sorafenib, Panobinostat and AZD6244 were associated with OXSM in the CCLE database. Moreover, the possible targets of PLX4720, PD−0325901 and AZD6244 might be RAB7A (Figure 8).

## Discussion

Disulfidptosis is a newly defined mode of programmed cell death. Mechanistically, under the condition of glucose depletion, high SLC7A11 expression leads to excessive disulfide bond formation within cytoskeletal proteins, resulting in actin network collapse and cell death (Liu X. et al., 2023). In SLC7A11-high tumor cells, pharmacological inhibition of glucose uptake mediated disulfidptosis, revealing a promising therapeutic strategy for cancer (Liu X. et al., 2023). However, the development of disulfidptosis-targeted therapies remains challenging due to an incomplete understanding of its role in tumor progression and drug sensitivity. Our study performed a bioinformatic analysis of disulfidptosis-related genes in CC patients and established a 5-DRGs prognostic model (RAB7A, SLC7A11, INF2, FLNA, and OXSM) which was used to calculate each patient’s risk score. The model showed excellent performance in risk discrimination and predicting prognosis. The model effectively stratified CC patients into high- and low-risk groups with different overall survival outcomes in both the TCGA training cohort and the GSE39582 validation cohort. The Kaplan–Meier curves revealed that patients in the low-risk group had prolonged survival compared to those in the high-risk group (P < 0.05, Figures 3C,D), indicating the model’s robust capacity for risk discrimination. Multivariate Cox regression analysis confirmed that the model served as an independent prognostic factor (HR = 2.25, P < 0.05), after adjusting for TNM stage and sex (Figures 3I,J). The model was not influenced by gender but demonstrated significant risk discrimination among various TNM stages (Figure 3N). In the external validation cohort (GSE39582), the five-gene model demonstrated only marginal discrimination, with time-dependent AUCs ranging from 0.54 to 0.56 (Figure 3H). Given that these values are close to 0.5, the signature appears to have limited predictive performance in this dataset, underscoring constraints in generalizability. Several factors may contribute to this drop in AUC compared with the discovery cohort, including inter-cohort heterogeneity (patient composition, treatment patterns, and follow-up), platform and preprocessing differences in gene-expression measurements, and potential overfitting during feature selection in retrospective datasets. Therefore, our signature should be interpreted as an exploratory prognostic signal.

To further clarify the differences between the two disulfidptosis-related subtypes of immune infiltrating cells, we used the ESTIMATE algorithm to perform the analysis. 13 immune cells have significantly different abundance. In the low-risk group, anti-tumor immune cells like CD56bright NK cell (Wagner et al., 2017), γδ T cell (Saura-Esteller et al., 2022), plasmacytoid dendritic cell (Reizis, 2019) (pDCs)and type 17 T helper cell (Son et al., 2023) were enriched in the TME of low risk patients. CD56 bright NK cells are primarily localized in lymph nodes, where they respond to cytokines such as IL-12 and IL-18 by secreting substantial amounts of IFN-γ. This response enhances the antigen-presenting capacity of dendritic cells (DCs) and promotes cytotoxic T lymphocytes (CTLs) activation, thereby indirectly amplifying antitumor immunity (Masmoudi et al., 2025). Okada et al. indicated that in patients with stage II CC, a higher density of CD56bright NK cells in the lymph nodes was identified as an independent favorable prognostic factor, distinct from T stage, tumor differentiation, or lymphatic invasion (Okada et al., 2018). However, within the TME, CD56bright NK cells often exhibit impaired cytotoxic function despite their immunomodulatory potential. This dysfunction may be attributed to the high expression of targetable immune checkpoints, such as CXCR4 and IL-1R8 (an IL-1 receptor family member), which suppress the ability of CD56bright NK cells to eliminate tumor cells (Mikulak et al., 2025). Notably, IL-15 priming or the use of CXCR4/IL-1R8 inhibitors can enhance the antitumor capacity of CD56bright NK cells (Wagner et al., 2017). As a unique lymphocyte population bridging innate and adaptive immunity, γδ T cells recognize tumor stress ligands independent of antigen presentation by human leukocyte antigen (HLA) molecules and exhibit antitumor cytotoxicity. Their infiltration is associated with favorable prognosis across multiple cancer types (Saura-Esteller et al., 2022). Although γδ T cells demonstrate functional plasticity in tumor immunity, under specific conditions, γδ T cells in tumor immunity can be driven toward IFN-γ^+^ and Th1-like subsets. These effector cells subsequently amplify immune responses by directly activating DCs and CD8^+^ CTLs. (Miyashita et al., 2021). Furthermore, anti-PD-1/CTLA-4 therapy significantly increases the number of tumor-infiltrating γδ T cells and enhances their antitumor efficacy (de Vries et al., 2023). In our study, increased infiltration of CD56bright NK cells and γδ T cells were observed in low-risk patients. These findings suggest a potential therapeutic strategy: combining cytokine IL-2/15 priming with ICIs or conventional therapies such as chemotherapy/radiotherapy may fully unleash the antitumor potential of CD56 bright NK cells and γδ T cells. Such an approach could potentially overcome resistance to ICIs, particularly in malignancies where these cells are enriched but functionally suppressed (Wagner et al., 2017; Miyashita et al., 2021). We also found that in CC, the low-risk group might respond better to CTLA4 treatment compared with the high-risk group in both training cohort and validation cohort identifying low-risk CC patients as potential beneficiaries of immune checkpoint inhibitor therapies. For most cancer histologies, an association between higher TMB and improved survival was observed (Samstein et al., 2019). The high-risk group exhibited a lower TMB, which is indicative of a poor prognosis and potentially impaired immunogenicity in CC. This finding suggests that tumors with low TMB may evade immune surveillance. Consequently, a lower disulfidptosis-related DRGs risk score appears to reflect a more active antitumor immune state. Although immune infiltration analyses and TMB show certain trends, these findings are derived from computational analyses and further functional experimental validations are expected.

Mechanistically, the model genes are involved in key cellular processes related to disulfidptosis and in the tumor behavior of CC. It is known that high expression of SLC7A11 is one of the triggers of disulfide stress (Liu X. et al., 2023). A whole-genome CRISPR/Cas9 screening in SLC7A11-overexpressing 786-O cells under glucose-replete and -starved conditions was conducted and found that inactivation of SLC7A11 synergizes with glucose starvation to induce tumor cell death (Liu X. et al., 2023). High RAB7A expression strongly correlated with aggressive clinicopathological features—including larger tumor size, deeper local invasion (advanced T stage), positive lymph-node metastasis (N1/N2), distant metastasis, and older patient age. RAB7A protein is an independent prognostic factor for poor overall survival which is consistent with the conclusions we have drawn (Figures 3I,6A,B [Fig F6]) (Shan et al., 2025). Hu et al. demonstrated that OXSM participates in mitochondrial fatty-acid synthesis and may indirectly modulate disulfidptosis sensitivity by altering lipid metabolism or oxidative stress. Compared with normal colon tissue, OXSM is markedly downregulated in tumor tissue, and its low expression is significantly associated with shorter overall survival, identifying it as a key protective factor. Analogous results were observed in our own research (Figures 3I,6E,F [Fig F6]) (Hu et al., 2023). In CC, FLNA, the downstream gene of WTAP, can inhibit autophagy (Huang et al., 2023). It is most frequently mutated in colorectal cancer (43%) and other cancers (Liu and Tang, 2023). One of the selective autophagy-targeted organelles is mitochondria in which INF2 as a risk factor from this research mediates the oncogenic development by mitochondrial fission (Jin et al., 2017; Li et al., 2019; Ding et al., 2024), although the role of INF2 in CC is currently unknown.

To ensure our findings were robust, we designed biological experiments to validate the key differentially expressed genes, going a step further than earlier studies in this area. Of the 5 DRGs, OXSM and RAB7A have rarely been reported on CC in previous research. To date, research on OXSM has been limited. Available studies have shown that low OXSM expression is associated with an unfavorable prognosis in ovarian and colon cancer patients. OXSM downregulation markedly increases cell proliferation and migration. These results indicate that OXSM plays a role in inhibiting cancer progression (Duan et al., 2023; Hu et al., 2023). RAB7A is involved in a variety of cancer progression processes, including sustaining proliferative signaling, activating invasion and metastasis, reprogramming energy metabolism, and evading immune destruction (Guerra and Bucci, 2019). There are numerous reports in the literature indicating RAB7A as a lead actor of cancer progression (Guerra and Bucci, 2016; Liu Y. et al., 2023; Ju et al., 2024). For example, increased expression of vimentin—an intermediate filament protein and one of the effectors of RAB7A—significantly enhances the migratory and invasive abilities of cancer cells (Messica et al., 2017). Notably, high expression of RAB7A is associated with the oncogenic mitogen-activated protein kinase (MAPK) signaling pathway. This pathway is highly activated in a variety of tumors, and many of its components have been identified as oncogenes (Liu et al., 2022; Drosten and Barbacid, 2020). In our study, OXSM and RAB7A expression was significantly different between tumor stages, especially between Stage I, Stage II and III. Enhanced expression of OXSM occurred in Stage 0 suggesting that it may be a cancer relieving molecule. It is another direction for RAB7A investigation. While our TMA results in the protein aspect reveal RAB7A is significantly upregulated in tumors and associated with poor prognosis, OXSM shows contrasting downregulation and functions as a protective factor (Figure 7E). Compared with the roles of RAB7A and OXSM genes in tumorigenesis reported in relevant literature, the results are consistent with our research findings. Further, PLX4720 and PD−0325901 are possible targeted therapeutic agents for RAB7A (Figure 8). PLX4720 is an inhibitor of Serine/threonine-protein kinase B-raf, which is involved in transducing mitogenic signals from the cell membrane to the nucleus. PD-0325901, also known as Mirdametinib, is an inhibitor of dual-specificity mitogen-activated protein kinase kinase (MAP2K). Both drugs act through the same molecular pathway as RAB7A in tumor development and progression—the MAPK signaling pathway—thus establishing a coherent link at the molecular mechanism level (Drosten and Barbacid, 2020).

Nevertheless, there still are several limitations in our study. Our model is primarily constructed using retrospective transcriptomic and clinical data from public databases. These data may suffer from selection bias, incomplete information recording, cohort heterogeneity, and batch effects across different cohorts, which may partially explain why RAB7A expression do not reach statistical significance in the GSE39582 validation cohort. The model relies on bulk tumor tissue gene expression data, which may not reflect CC heterogeneity and dynamics. Additionally, no adjustments are made for other confounding factors, such as specific treatments (chemotherapy, targeted therapy, etc.), the tumor immune microenvironment (e.g., spatial distribution of specific immune cells), and lifestyle differences—all of which may independently influence patient survival. Prospective studies are warranted to validate the clinical utility of our model. The current research conclusions are primarily derived from bioinformatics analysis. Although we preliminarily validated the association between the expression of RAB7A and OXSM and prognosis at the protein level using TMA, in-depth functional experiments are lacking to elucidate the specific molecular mechanisms by which these two key genes regulate disulfidptosis and influence tumor progression or the immune microenvironment in CC. The proposed potential immunotherapy strategies and the development of targeted therapeutic drugs for CC patients with high RAB7A expression are based on computational predictions and reasonable extrapolations, representing potential research directions rather than completed components of this study.

## Conclusion

This article constructed and validated a five-gene disulfidptosis-related signature as an independent prognostic indicator for CC patients (RAB7A, SLC7A11, INF2, FLNA, and OXSM). The risk score thus established can independently predict the survival and immunotherapy benefit of patients with CC. Through experimental validation, we demonstrated RAB7A as an important risk factor and extracted its potential target drugs (PLX4720 and PD−0325901) from the database. The above results could provide a basis for future pharmacological and immunotherapeutic study.

## Data Availability

The datasets presented in this study can be found in online repositories. The names of the repository/repositories and accession number(s) can be found in the article/Supplementary Material.
